# DPRESS: Localizing estimates of predictive uncertainty

**DOI:** 10.1186/1758-2946-1-11

**Published:** 2009-07-14

**Authors:** Robert D Clark

**Affiliations:** 1 Biochemical Infometrics, 827 Renee Lane, Creve Coeur MO 63141, USA; 2 School of Informatics, Indiana University, 901 E 10th St, Bloomington IN 47408, USA

## Abstract

**Background:**

The need to have a quantitative estimate of the uncertainty of prediction for QSAR models is steadily increasing, in part because such predictions are being widely distributed as tabulated values disconnected from the models used to generate them. Classical statistical theory assumes that the error in the population being modeled is independent and identically distributed (IID), but this is often not actually the case. Such inhomogeneous error (heteroskedasticity) can be addressed by providing an individualized estimate of predictive uncertainty for each particular new object *u*: the standard error of prediction *s*_u _can be estimated as the non-cross-validated error *s*_t* _for the closest object t* in the training set adjusted for its separation *d *from *u *in the descriptor space relative to the size of the training set.

The predictive uncertainty factor *γ*_t* _is obtained by distributing the internal predictive error sum of squares across objects in the training set based on the distances between them, hence the acronym: *D*istributed *PR*edictive *E*rror *S*um of *S*quares (DPRESS). Note that *s*_t* _and *γ*_t*_are characteristic of each training set compound contributing to the model of interest.

**Results:**

The method was applied to partial least-squares models built using 2D (molecular hologram) or 3D (molecular field) descriptors applied to mid-sized training sets (*N *= 75) drawn from a large (*N *= 304), well-characterized pool of cyclooxygenase inhibitors. The observed variation in predictive error for the external 229 compound test sets was compared with the uncertainty estimates from DPRESS. Good qualitative and quantitative agreement was seen between the distributions of predictive error observed and those predicted using DPRESS. Inclusion of the distance-dependent term was essential to getting good agreement between the estimated uncertainties and the observed distributions of predictive error. The uncertainty estimates derived by DPRESS were conservative even when the training set was biased, but not excessively so.

**Conclusion:**

DPRESS is a straightforward and powerful way to reliably estimate individual predictive uncertainties for compounds outside the training set based on their distance to the training set and the internal predictive uncertainty associated with its nearest neighbor in that set. It represents a sample-based, *a posteriori *approach to defining applicability domains in terms of localized uncertainty.

## Background

Early work on quantitative structure-activity relationships (QSAR) was primarily concerned with relating select physical properties to *in vivo *biological activity [1,2]. Ordinary least squares regression (multiple linear regression) was the analytical tool of choice, and the statistical questions addressed focused on whether a particular descriptor was significant or not. QSAR methods soon evolved, however, into being ways of identifying optimal physical properties rather than simply trends, a shift accomplished by fitting to quadratic and bilinear equations. This development was spurred in no small part by the desire to identify optimal octanol/water partition coefficients (logP), generally in pursuit of optimal *in vivo *activity.

The focus for pharmaceutical drug discovery subsequently shifted from *in vivo *testing to *in vitro *evaluation of interactions between candidate ligands and isolated enzymes or receptors. This change brought with it a shift of descriptors from measurable properties of compounds to computationally estimated properties of molecules, with the calculations in question often being based on (sub)structural descriptors. The next step was to take descriptors into account that were based on molecular structure but were not themselves measurable physical properties. Often these were more or less local in nature, and the purposes of doing the analysis shifted from identifying significant underlying relationships to the descriptors to identifying optimal substituents or substitution patterns. Interest in artificial neural networks (ANNs) [3] and partial least squares with projection onto latent structures (PLS) [4] as analytical tools increased at the same time. Questions related to validity of the model as a whole took center stage as the number of descriptors available proliferated [5,6], followed closely by a strong interest in predictivity and how best to establish applicability domains [7-15].

Today, however, the overall statistical properties of a particular QSAR are less relevant to medicinal chemists or environmental regulatory agencies. Recent pressure to simultaneously reduce clinical failures, ensure the safety of bulk chemicals [16-18] and reduce testing on animals have led to an increasing reliance on models for predicting off-target biological effects and toxicity. This use of QSAR models entails applications to more structurally diverse compounds, but it also changes the relative importance of different kinds of mistakes. If a structure is predicted to have a much higher affinity for the target than it actually does, the cost to a lead optimization program is limited to the synthetic resources wasted on that particular structure. Even that cost is mitigated if something useful was learned about the underlying structure-activity relationship (SAR) in the process. Such a false positive error in predictive toxicology, however, may mean that a life-saving (and profitable) drug never gets commercialized. Compounds mistakenly predicted to be inactive – false negatives – represent a missed opportunity in the context of lead optimization, but they have the potential to be downright catastrophic (and ruinous) in the context of predictive toxicology.

Such considerations put a premium on being able to make a quantitative estimate of how reliable an *individual *prediction obtained from a given model is. What is more, answers to the question, "How reliable are the predictions about this *particular *molecule that I am considering for synthesis, clinical evaluation or registration?" are often most relevant for extrapolations to structures near the "outside" edges of the descriptor space defined by the training set. Hence, to be of practical use, constraints on applicability domains need to be "soft" – i.e., increase with distance from the descriptor space covered by the training set – but "hard" enough to indicate just how far outside the training set one can safely expect to go. They also need to provide a robust quantitative estimate of predictive reliability that is sensitive to local variations in the descriptor space. This paper presents a novel methodology for doing exactly that based on how close a new compound is to those in the training set and the distribution of internal predictive error across compounds in that set.

### Classical statistical theory

The underlying model for linear regression on a vector **X **of *p *independent variables is reflected in Eq. 1, wherein *Y *is the response variable of interest, *μ*_Y _is the population mean of *Y*, **β **is a vector representing the sensitivities of *Y *to changes in **X**, and **x **is a vector of deviations in **X **from the population centroid **μ**_X_.(1)

As indicated in Eq. 1, the error *ε *is assumed to be normally distributed with mean 0 and a standard deviation *σ*_X_. Best linear unbiased estimators (BLUEs) for the various parameters in Eq. 1 can be calculated from a sample *T*_0 _of *n *observations (in QSAR, compounds) drawn from the full population, provided several preconditions are met [19]:

**1**. the strict linear dependence of *Y *on **X **set out in Eq. 1 applies across the population;

**2**. the sample is random and unbiased;

**3**. the descriptors contributing to **X **are mutually independent in a statistical sense; and

**4**. the error distribution *ε *is *homoskedastic *and independent of **X **and *Y *– i.e., its standard deviation is the same everywhere in the descriptor space, so *σ*_X _= *σ *for all **X**.

The corresponding regression estimators for each individual observation *i *and the overall standard error of regression *s*_FIT_are then given as shown in Eqs. 2 and 3.(2)(3)

where  is the mean value of *Y *for the sample; **x**_i _= **X**_i _- **X**_0_, with **X**_0 _being the sample centroid for **X**; and  is the predicted value of *Y *at **X**_i _[19]. Note that *s*_FIT _is greater than the root mean square error (RMSE); this is because the means  and **X**_0 _and the calculated coefficient vector **b **are themselves estimates that are subject to sampling error, with 1 and *p *degrees of freedom, respectively.

Under these assumptions, the potential error in estimating *Y *increases as one moves away from the centroid **X**_0_. As a result, the uncertainty *s*_u _in predicting the value of *Y *at some new ("unknown") value **X**_u _is generally greater than *s*_FIT_. In fact, under the assumptions given above [19]:(4)

where *s*_u _is the expected standard error of prediction (uncertainty) for the new observation *u *and *n *is the number of training set observations *t *used to build the model. The Mahalanobis distances *d*_0, u _and *d*_0, t _are measured in the model space defined by **b**, i.e., they are weighted Euclidean distances between the centroid **X**_0 _of the descriptor matrix for the training set and the vectors **X**_u _and **X**_t_, respectively.

The rationale behind the "extra" terms in Eq. 4 is straightforward. For any random sample, the error involved in using  as an estimate of *μ*_Y _is inversely proportional to *n *– hence the 1/*n *term in Eq. 4. In addition, the accuracy with which *β *is estimated by **b **is inversely proportional to how thoroughly **X **is sampled by the training set, but how much difference that makes to the error is directly proportional to the distance *d*_0, u _between **X**_u _and **X**_0 _in the model space. Together these countervailing effects of variation in **X **account for the second term within the outer brackets.

### Dealing with violated assumptions

The value of *s*_u _produced by Eq. 4 is a best linear unbiased estimator of *σ*_u _– *provided the assumptions underlying its derivation hold*. Unfortunately, one or more of those assumptions are violated in most QSAR applications. In particular:

**1**. the dependence of *Y *on **X **rarely fits the prescribed function perfectly, linear or otherwise;

**2**. the training set used is usually a non-random sample, its selection biased by matters of historical accident and convenience that reflect the historical trajectory of the synthesis program that motivated the analysis;

**3**. the descriptors contributing to **X **are often correlated to a greater or lesser degree and hence are not independent variables in the statistical sense (correlation implies lack of independence, but the inverse is not true: lack of correlation does not imply statistical independence); and

**4**. *ε *is usually heteroskedastic – its standard deviation *σ*_X _is often different in different regions of the descriptor space.

Most or all of the assumptions are, in fact, explicitly violated when ANNs, PLS, variable selection, quadratic regression, or bilinear regression techniques are applied, with the result that *s*_FIT _and the estimator given by Eq. 4 underestimate the actual uncertainty of prediction, often drastically.

Several groups have derived theoretical variations of Eq. 4 for use with PLS and principal component analysis (PCA) that seek to address departures from ideality [20-22]. Unfortunately, subsequent work has demonstrated that these methods are often not robust when applied in realistic situations [23].

An alternative, completely empirical approach to assessing aggregate predictive uncertainty is cross-validation, in which each compound in the training set is held back in turn [24]. The value of *Y *for the held-back compound is then predicted using a model built from the other *n *- 1 compounds in the training subset *T*_u _= *T*_0 _- {*u*}. In parallel to Eq. 3, the standard error of cross-validation *s*_CV _is calculated from the predictive error sum of squares (PRESS) according to equation 5:(5)

where  is the value of *Y *predicted by applying the reduced model built from the *n *- 1 compounds in training subset *T*_u _to **X**_u _and  is the corresponding predictive error. The summation is indexed across *u *to emphasize that prediction is external to the training subset used in each case. Here *p *represents the number of PLS components included in the model rather than the number of descriptors.

Cross-validation statistics were originally employed in PLS solely as a way to determine an optimal model complexity, a role for which the classical goodness-of-fit measure *r*^2 ^used in ordinary least squares is unsuited [24]. It has since come to widely used to assess predictivity, however. This use is unfortunate, in that a poorly predictive model will have a high *s*_CV _and a low *q*^2^, but the converse may or may not be true: good cross-validation statistics may be due to redundancies in the training set rather than truly robust predictive performance [25-28]. Some workers prefer to use "leave-some-out" cross-validation – in which several compounds are held back together – to address this problem. Nonetheless, the LOO standard error is the best estimate of the full model's predictivity for each individual compound in the training set [29], which makes it is a reasonable starting point for estimating a model's predictive reliability for structures occupying nearby points in the descriptor space.

Violation **4 **– that error is not identically and independently distributed across compounds – is especially problematic for QSAR analyses. In one recently described case in point, the variation in predictive error was clearly correlated with one of the two descriptors being used [7]. If that is true when many descriptors are involved (as is the case for PLS), the *overall *variability in predictive error should be similar across the full range of *Y*. Such a distribution of error is, in fact, often seen in place of the quadratically increasing spread implied by Eq. 4 [30]. This makes it all too easy to make the unjustified leap to the unjustified conclusion that the aggregate predictive uncertainty – typically *s*_CV _or the root mean square error of prediction for an external test set (RMSEP or *s*_PRED_) – is a reliable indicator of the level of uncertainty associated with *individual *predictions: independence from *Y *does not imply independence from **X**.

### Partitioning the PRESS

The increasing reliance of drug developers on tabulations of predicted properties makes getting accurate estimates of the uncertainty *σ*_u _for individual predictions critically important. Unfortunately, it is rarely if ever possible to construct a unified global model for the dependence of *σ*_u _on **X**. It is neither necessary nor even desirable to do so, however. A better approach is to shift from the classical, descriptor-based view of regression to a sample-based formalism such as that used in the SAMPLS algorithm [31]. This algorithm exploits the fact that Eq. 2 can be recast as Eq. 6 without loss of generality:(6)

where **c**_t, i _= [*x*_t1_*x*_i1 _*x*_t2_*x*_i2 _... *x*_tp_*x*_ip_] is the covariance between **x**_t _and **x**_i _and **v**_t _is a weight vector that is specific to compound *t*. Basically, Eq. 6 says that activity can be expressed as a linear function of the similarities of each compound to each of the other compounds in the training set. This suggests that the observed predictive error *e*_u _can be cast as a sum of contributions from each compound in the training set that increases with similarity to those compounds, which is consistent with the observation that predictive error tends to increase with distance from – i.e., tends to decrease with increasing similarity to – compounds in the training set [11,12,15]. If t* is the closest (i.e., the most similar) such compound, its standard error (*s*_t*_) is a reasonable first approximation to the predictive error *s*_u _for a new compound. In most QSAR applications, a single response value *Y*_t _is assigned to each compound in the training set, so the best estimate of *s*_t _is simply |*e*_t_|, where *e*_t _is the deviation seen for *t *in the full, non-cross-validated model, i.e., the residual error of fitting.

Though the "true" dependence of predictive uncertainty on the Euclidean distance *d*_t*, u _from t* is unknown, its dependence on distance can likely be approximated by a Taylor expansion in which all but the first, linear term in *d *is dropped. Taken together, these considerations yield the estimator defined by Eq. 7:(7)

where *d*_00 _is the length of the vector **x**_00 _defined by the standard deviations of the descriptors; *d*_00 _= 1 when descriptors have been centered and autoscaled, as was the case here.

**Figure 1:**
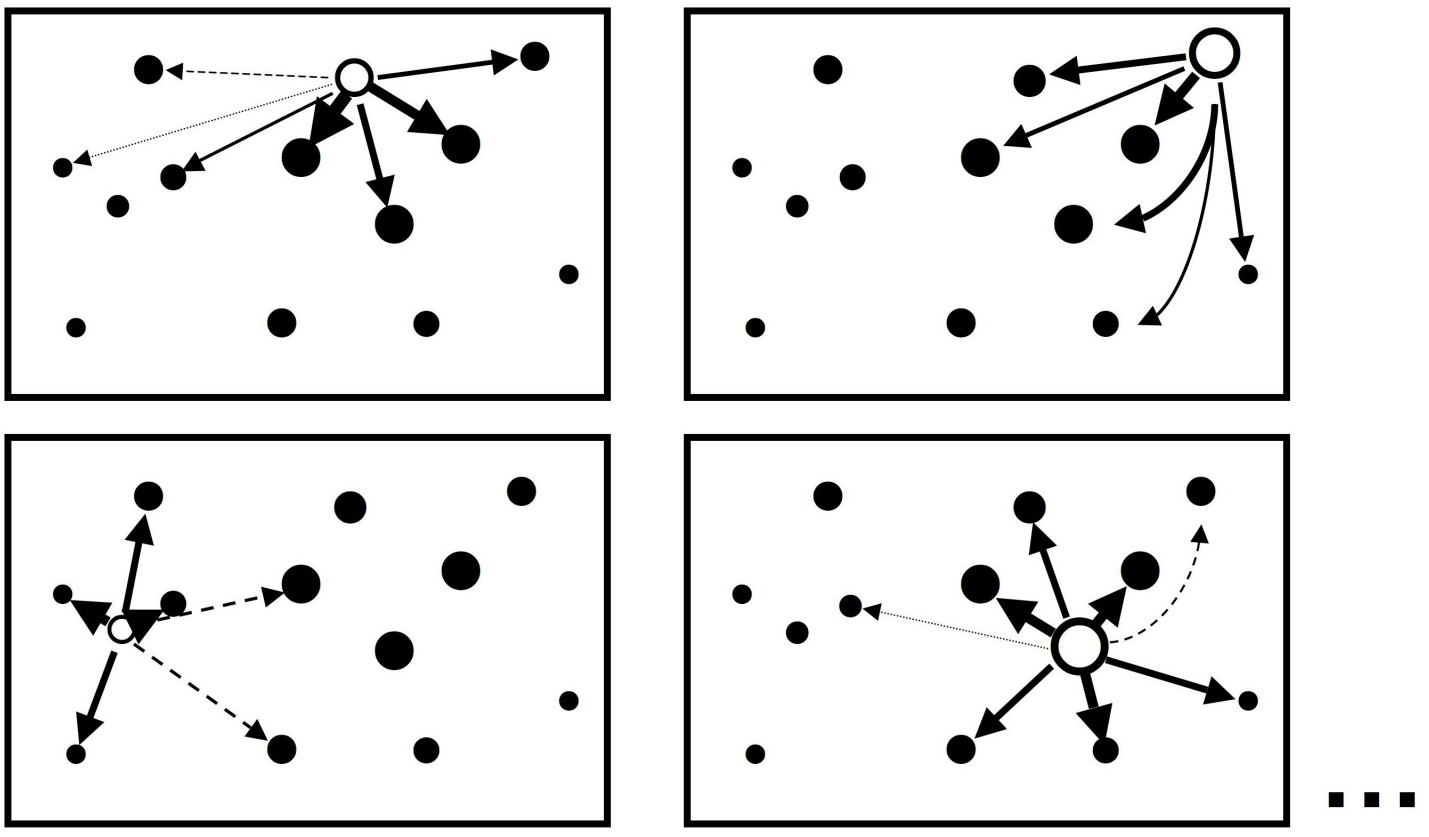
**Schematic representation of predictive error distribution in DPRESS**. The arrow weights indicate how much of the error made in predicting the response for the held-out compound (open symbol) is distributed among the compounds in the training set (solid symbols) when calculating the scaling factors *γ*_t_. The data set is comprised of 13 observations in a two-dimensional descriptor space. Each panel represents one of the 13 separate analyses that make up the full leave-one-out (LOO) cross-validation run; only four of the 13 are shown.

The problem then becomes one of estimating the predictive error *γ*_t _associated with each compound *t *in the training set. PLS tends to overfit, so this term is likely to be greater than *s*_t*_; otherwise Eq. 7 would parallel Eq. 4 exactly, except for the loss of the 1/*n *aggregation term within the brackets. Instead, one can turn to the squared predictive errors collected during cross-validation. In the calculation of the aggregate predictive uncertainty *s*_CV _(Eq. 5), these are lumped into a single sum – the PRESS. If, however, one assumes that contributions from nearby training set compounds dominate the predictive error and, further, that the value of *γ *will be comparable for the training subset compounds closest to each individual compound *u*, the contribution  that cross-validation of the *i*^th ^compound makes to the PRESS can more appropriately be distributed across the training subset in inverse proportion to the distances between **X**_i _and the *n *- 1 compounds used to predict *Y*_i _(Eq. 8 and Fig. 1). A similar approach is taken to distributing response variance across the various sources of deviation from the mean in classical analysis of variance (ANOVA).(8)

The normalization factor *α*_i _in Eq. 9 is necessary to ensure that the distribution is a partition – i.e., that the contributions from the cross-validation step in which compound *i *was set aside sum to the observed cross-validation error in prediction .(9)

A small constant (1/*n*) needs to be included to prevent the reciprocal from "exploding" at small distances. Basically, it dictates the distance at which error is expected to distribute evenly. The choice of this particular value is somewhat arbitrary, but 1/n works well and nicely accommodates the tendency of data points to get closer together as the training set gets larger. Taken together, Eqs. 7–9 define the *D*istributed *PR*edictive *E*rror *S*um of *S*quares (DPRESS) approach to estimating predictive uncertainty.

## Results

The suitability of DPRESS or any other quantitative model of predictive uncertainty is best evaluated by applying it to experimental QSAR data sets. Here, DPRESS is tested against PLS models obtained using a 3D descriptor (comparative molecular field analysis, or CoMFA [32-34]) and a 2D descriptor (hologram QSAR, or HQSAR [35-37]). A large data set (*N *= 304) was used to insure that the number of compounds held back to evaluate external predictivity was much greater than the numbers needed to train a reasonably robust model.

### The data set

**Figure 2:**
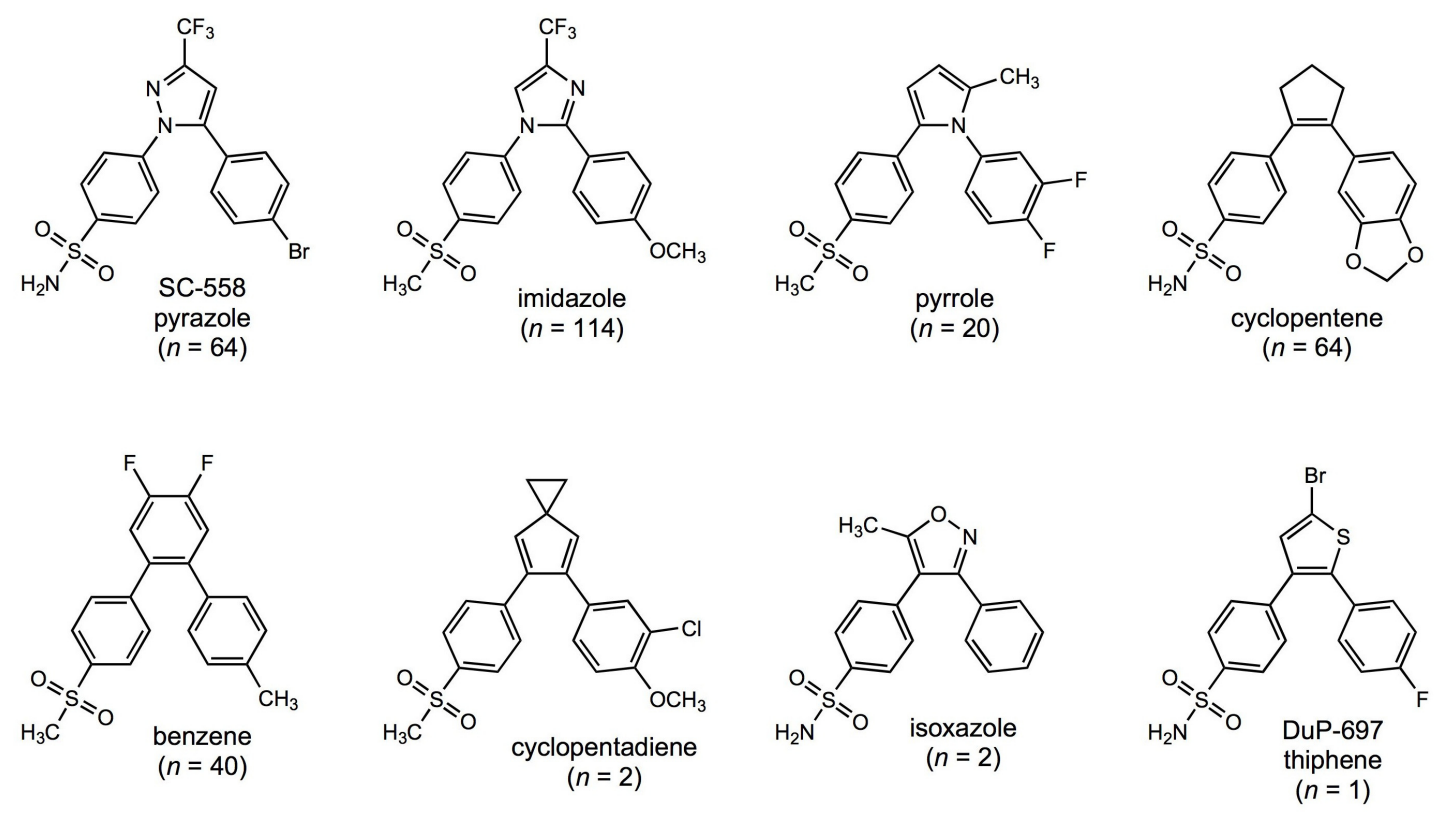
**Representative examples from the five major and three minor structural classes included in the COX-2 data set**. The number of members in each class are indicated in parentheses. Each of the five major classes includes both sulfonyl and sulfamoyl analogs.

The set of structurally diverse cyclooxygenase inhibitors examined here was originally compiled by Chavatte et al. [38]. It includes data on five major and three minor structural classes (Fig. 2) of inhibitors of the inducible form of the enzyme (COX-2). This data set is attractive because the target has been a major focus of research on anti-inflammatory drugs and because it combines substantial structural variation with a few key shared elements such as the distal sulfonyl (SO_2_CH_3_) or sulfamoyl (SO_2_NH_2_) group. In addition, regression models based on this data set are well-characterized in terms of predictive robustness [25,28] and with respect to variations in how training subsets are selected [39]. Finally, the uneven representation of the different core structures reflects a sampling bias that is typical of the data sets used to build QSARs.

**Figure 3:**
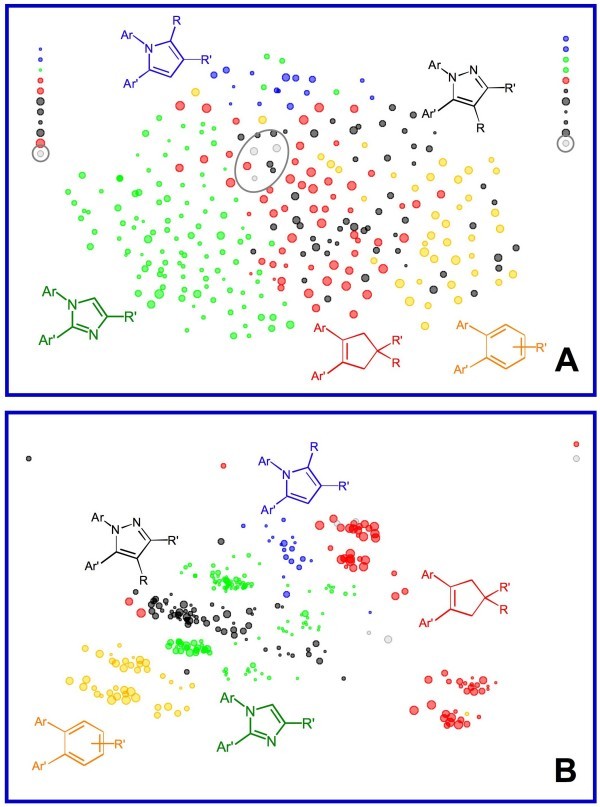
**The distribution of activity across descriptor spaces for compounds in the COX-2 data set**. Symbols are color-coded by structural class and symbol sizes are proportional to the negative common logarithm of the potency (pIC50). Compounds falling into the three minor classes (cyclopentadienes, isoxazoles and thiophene DuP-697) are indicated in gray. Points in the vertical "hedges" at the top left and top right of the plots represent singletons that are too dissimilar to any other compound to be placed meaningfully within the eNLM. (**A**) Projection obtained by applying embedded non-linear mapping (eNLM) to the Euclidean distance matrix calculated from steric and electrostatic fields. Points reprsenting compounds from the minor classes are circled. (**B**) Projection obtained by applying eNLM to the Euclidean distance matrix calculated from molecular holograms hashed to a length of 353. See the **Methods **section for details.

Fig. 3A shows how activity is distributed across the various structural classes when the compounds in the data set are projected into two dimensions using embedded non-linear mapping [40,41] based on the similarity in their molecular fields: symbols are colored by structural class and sized by activity. Clearly, no one structural class has a monopoly on high activity. Fig. 3B shows the distribution of activity across the descriptor space defined by the compounds' molecular holograms. Molecular fields are 3D descriptors, which are more generalized than holograms – 2D descriptors derived from substructure counts. The more literal character of holograms leads to smaller distances between inhibitors within classes relative to the distances between classes, which accounts for the greater between-class resolution in Fig. 3B. It also accounts for the fact that the sulfonyl and sulfamoyl subclasses are cleanly separated in the hologram space (Fig. 3B) but not in the space defined by the corresponding molecular fields (Fig. 3A).

The main goal of the work reported here was to see how well local estimates of predictive error obtained by DPRESS reflect the actual distribution of predictive error across the descriptor space. Simple random sampling produces a biased training set because, as in most such data sets, the major structural classes are not evenly represented (Fig. 2). Therefore diverse but representative ("boosted" [39]) training sets were generated by independently drawing five training (sub)sets of 75 compounds from the full set using optimizable *k*-dissimilarity (OptiSim) selection [39,42,43]. Models based on those training sets were then used to predict the activities of the 229 inhibitors not used to construct them. Three additional training sets were drawn at random, only one of which gave acceptable internal cross-validation statistics. Representation in the full data set is biased, so such simple random subsets are biased as well. The results obtained using that training set (set *R*) are included here to illustrate the effect of sampling bias due to structural redundancy [39,44,45].

### CoMFA models

The optimal number of components p* for the CoMFA models obtained for the boosted training sets ranged from three to seven. It is not appropriate to compare models that differ in complexity directly, however, so a consensus complexity of *p *= 6 was used in all cases. The corresponding leave-one-out (LOO) cross-validated standard errors (*s*_CV_) ranged from 0.681 to 0.762, corresponding to internal predictivities (*q*^2^) of 0.537 to 0.337. The non-cross-validated models exhibited standard errors of regression (*s*_FIT_) ranging from 0.279 to 0.398, corresponding to *r*^2 ^values between 0.901 and 0.827. Calculating the root mean square error for external predictions yielded *s*_PRED _= 0.633 to 0.655 – i.e., the internal cross-validated error underestimated the overall accuracy of external prediction somewhat.

In contrast, the biased training set *R *yielded a cross-validated standard error (*s*_CV_) of 0.489, corresponding to a *q*^2 ^of 0.696. The overall goodness-of-fit statistics for the non-cross-validated model were *s*_FIT _= 0.279 and *r*^2 ^= 0.901. As expected, however, the predictive performance on those compounds not in *R *was substantially worse than that of the boosted training sets, with *s*_PRED _= 0.744.

**Figure 4:**
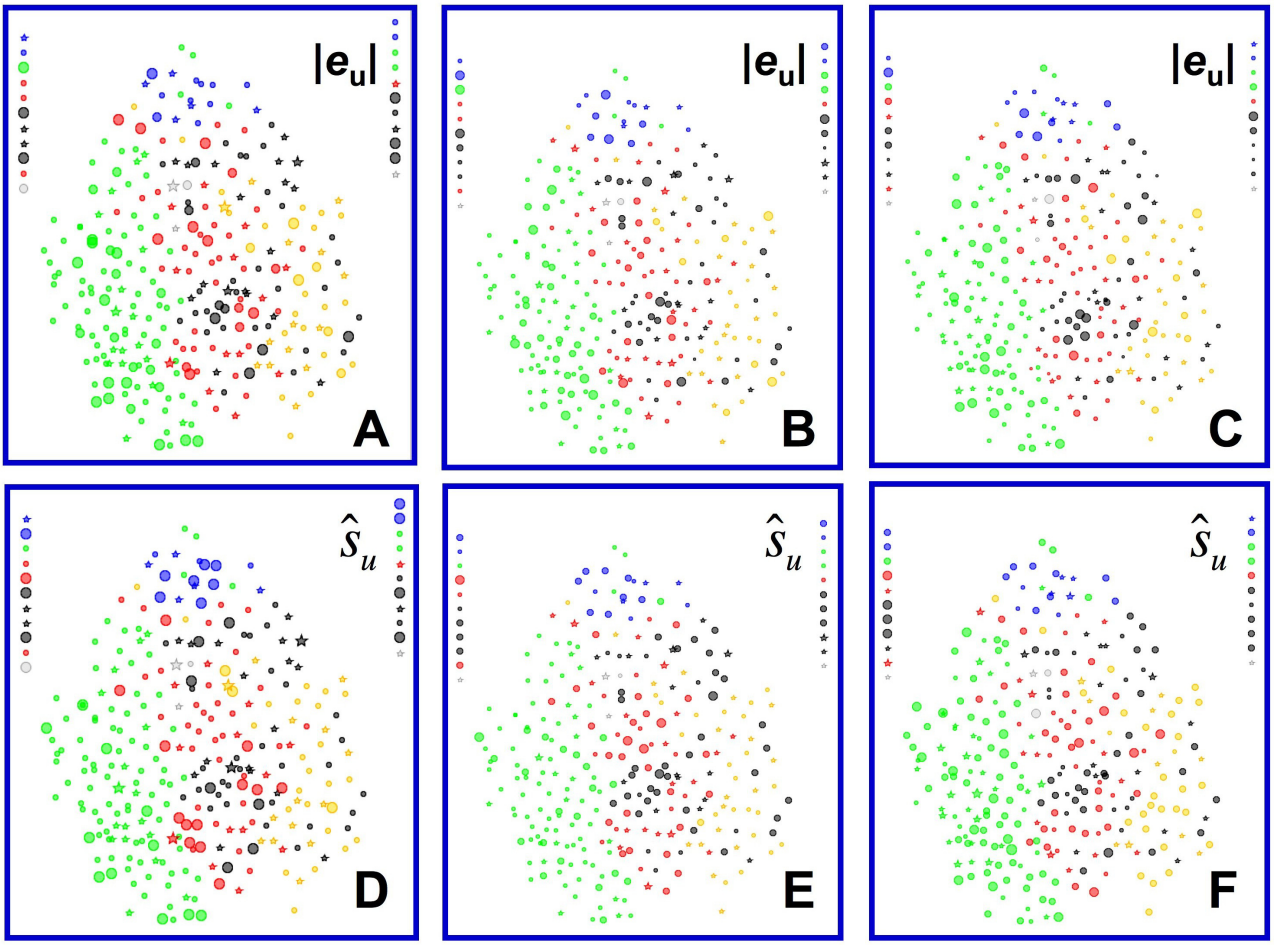
**Distribution of observed absolute errors and uncertainties predicted by DPRESS for three different CoMFA models**. Projection parameters and color coding are the same as in Fig. 3A except that the horizontal dimension has been compressed somewhat. Symbol size is proportional to the magnitude of the observed error or predicted uncertainty. Compounds from the respective training sets are represented by stars. (**A**) Observed absolute errors for boosted training set *A*, which had the best external predictive performance (*s*_PRED _= 0.633; *s*_CV _= 0.762). (**B**) Observed absolute errors for boosted training set *B*, which had the best internal predictive performance (*s*_CV _= 0.681; *s*_PRED _= 0.637). (**C**) Observed absolute errors for the biased training set (*s*_CV _= 0.489; *s*_PRED _= 0.744). (**D**) Predicted uncertainties for boosted training set *A*. (**E**) Predicted uncertainties for boosted training set *B*. (**F**) Predicted uncertainties for the biased training set *R*.

Fig. 4A shows the same projection as Fig. 3A, but here symbol sizes are based on the error in predicted pIC50 rather than on pIC50 itself. The top panels in Fig. 4(A–C) show the distributions of the individual observed errors in predicted activity (|*e*|) across the descriptor space, whereas the bottom panels (D-F) show distributions of the corresponding predictive uncertainties () estimated using DPRESS. The leftmost panels (4A and 4D) were obtained for the model based on the boosted training set (set *A*) that had the lowest aggregate *external *predictive standard error (*s*_PRED_), whereas the middle panels (4B and 4E) are results for the boosted training set (set *B*) that had the lowest aggregate *internal *(cross-validated) predictive standard error (*s*_CV_) overall. The right-most panels (4C and 4F) display the results for the biased training set *R*.

Several conclusions can be drawn by comparing the distribution of errors to each other and to the distribution of activities. Firstly, though the distributions of observed predictive errors for the three models differ from one another (Fig. 4A vs 4B vs 4C), they resemble each other more than they resemble the distribution of activity itself (Fig. 3A). Secondly, the larger observed errors are not particularly concentrated among the singletons or at the edges of the descriptor space, as would be expected for the ordinary least squares distribution expected based on Eq. 6 and in most published approaches to establishing applicability domains. Thirdly, the distributions of predictive uncertainty seen for the boosted training sets are in good overall agreement with the observed errors with respect to the regions of descriptor space where the observed error is relatively high or low (Fig. 4D vs 4A and 4E vs 4B). Though somewhat less evident, the same is true for the model constructed using the biased training set *R *(Fig. 4F vs 4C). Finally, the smaller errors predicted by the boosted training with the better internal predictivity (Fig. 4E vs 4D) do seem to be realized in the localized errors actually observed (Fig. 4B vs 4A), even though this was not obvious in the aggregate statistics (*s*_PRED _= 0.637 and *s*_PRED _= 0.633, respectively).

**Figure 5:**
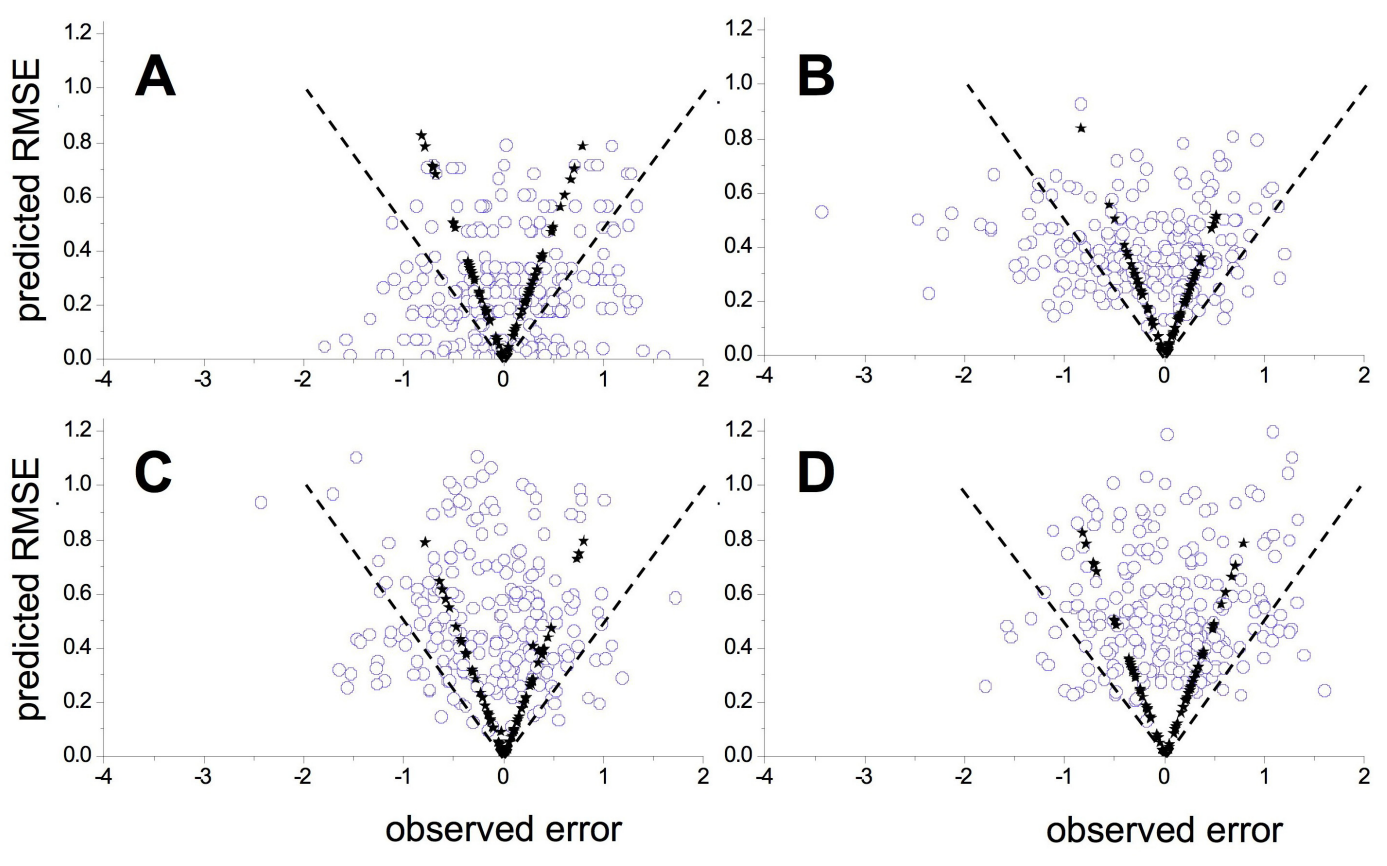
**Predictive uncertainty  as a function of the observed error for the CoMFA models**. Filled stars represent members of the training set and define the lines for  = |*e*_t_|. Dashed lines correspond to  = 2|*e*_u_|. (**A**) Results of setting *γ*_u _= 0 for all compounds. (**B**) Results for the model constructed from the biased training set *R*. (**C**) Results from boosted training set *A*. (**D**) Results for boosted training set *B*.

Interpretation of the plots shown in Fig. 4 is complicated because the uncertainty *s*_u _is a measure of the *spread *in predictive error at **X**_u_; the expected value of the error is still 0. If  is an accurate prediction of uncertainty, the magnitude of the observed error (|*e*_u_|) can be expected to be less than  about 68% of the time and to almost always (about 95% of the time) be less than 2. The plots in Fig. 5 – in which the predicted uncertainty (which is always positive) is shown as a function of the observed error (which can be positive or negative) represent a more quantitative way to see how well the predicted uncertainties track the spreads in error actually observed outside the training set.

Eq. 7 implies that  = |*e*_t_| for each member *t *in the training set. The corresponding points are represented by filled stars in each panel in Fig. 5, which therefore define the lines  = |*e*_u_|. Unbiased and normally distributed error should only fall outside these lines about 32% of the time and should fall outside the dotted lines corresponding to  = 2|*e*_u_| less than 5% of the time. This is clearly not the case when the cross-validated error for the most similar compound t* in the training set is taken as a direct estimate of , i.e., when *γ*_t _is set equal to 0 for all *t *(Fig. 5A). There are fewer unduly low predicted uncertainties for the biased training set *R*, but still more than would be expected by chance (Fig. 5B). Note that the bias evident in the model constructed from *R *comes mostly in the form of negative residuals, i.e., predicted activities that are larger than the observed activities. Such false positives account for most of the "extra" out-of-bounds errors seen in Fig. 5B. The distributions of errors for the boosted training sets are much better behaved; indeed, the predicted uncertainties are slightly more conservative than necessary for large errors in prediction (Fig. 5C and 5D).

### HQSAR

HQSAR analyses were carried out as a complement to the results obtained in the CoMFA studies described above. The 2D molecular holograms used were built up from the number of each kind of substructure comprised of between four and seven heavy atoms, the counts being mapped down into count vectors of various lengths by hashing [37]. HQSAR models were then constructed by applying PLS analysis to holograms of length 97, 151, 199, 257, 307 and 353. The optimal complexity for the full model (*N *= 304) was six components for all hash lengths. The *s*_CV _values obtained ranged from 0.609 to 0.640; the median and average were both 0.622. The value of *q*^2 ^ranged from 0.547 to 0.582, with a median of 0.564 and an average of 0.563. Based on these results, a hash length of 353 (*s*_CV _= 0.609 and *q*^2 ^= 0.582) was chosen for evaluating the behavior of the various training sets. The corresponding non-cross-validated analysis gave *s*_FIT _= 0.527 and *r*^2 ^= 0.687.

The consensus optimal complexity across the boosted training subsets was five components, in keeping with the full data set's having nearly four times as many compounds and, therefore, containing substantially more information. The *s*_CV _values obtained ranged from 0.691 to 0.776 versus a value of 0.540 for the biased training set *R*; the respective *q*^2 ^values were 0.386 to 0.514 and 0.623. The *s*_PRED _for the boosted subsets ranged from 0.619 to 0.669 and the corresponding value for the biased subset was 0.735. Hence HQSAR performance followed the trend seen for CoMFA: cross-validation under-estimated the predictive error substantially for the biased subset (i.e., was overly optimistic about the extensibility of the model) and over-estimated the predictive error slightly for the boosted training sets. It differed in that it was the boosted training set *B *which gave the better external predictive performance.

**Figure 6:**
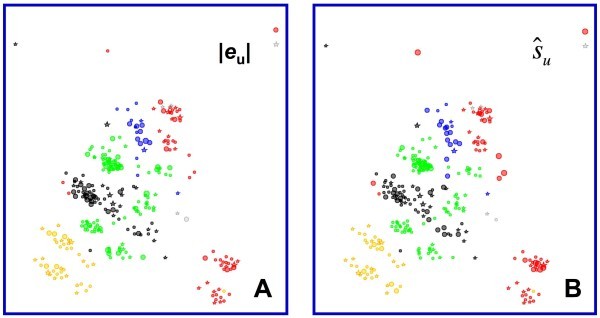
**Distribution of predictive error and uncertainty across the hologram descriptor space training set *A***. Stars correspond to compounds from the training set. Projection parameters and color coding by class are as indicated for Fig. 3B. Symbol sizes are proportional to magnitude. (**A**) Observed absolute predictive error. (**B**) Predicted uncertainty.

**Figure 7:**
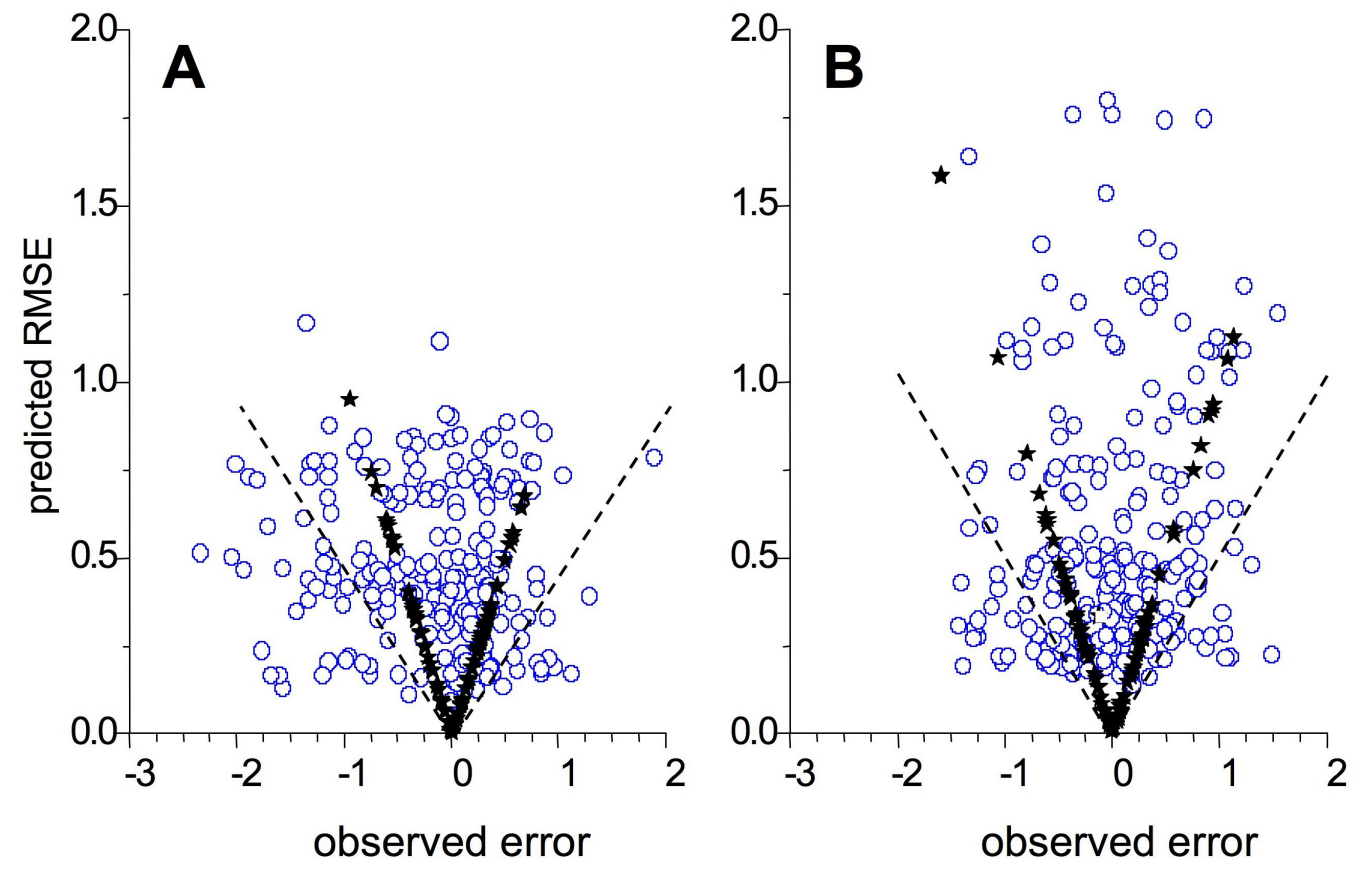
**Predicted uncertainty  as a function of the observed predictive error *e***. Filled stars correspond to compounds included in the test set, whereas open circles represent compounds in the test set. Dashed lines correspond to |e| = 2. (**A**) Results for the HQSAR model constructed from the biased training set *R*. (**B**) Results from boosted training set *B*.

The distribution of observed predictive errors and predicted uncertainties across the hologram descriptor space are shown in Fig. 6 for the model based on boosted training set *B*, and the corresponding plots of  as a function of *e*_u _are shown in Fig. 7. Note that the predicted uncertainties for the boosted HQSAR models were more conservative than those for the CoMFA models discussed above, with the result that the magnitudes of nearly all errors above 0.75 log units were less than the corresponding . This effect is probably a side-effect of the exaggerated separation between classes seen in the hologram space (Fig. 3B).

## Discussion

The degree to which any QSAR can be extended to compounds outside of the training set used to construct it is necessarily limited to some degree by the structural diversity of that training set. Some extensibility is necessary, however, if the QSAR is to be of use for something beyond mere rationalization of known activities. When only a few descriptors are being considered, it may be possible to restrict the applicability domain to "internal" regions in the descriptor space, but as the number of descriptors increases distinguishing compounds that lie "outside" the space defined by the training set from those that are "inside" becomes progressively less meaningful. Regardless of the complexity of the system, it is clear that one will often need to extend the applicability domain beyond the training set somehow. It is equally clear that this must be done cautiously, however, and that it would be desirable for the degree of caution to reflect the idiosyncrasies of the QSAR being examined. It would be particularly desirable to take local variations in the uncertainty of predictions into account, rather than trying to find a single acceptable distance to the model that is applicable across the entire descriptor space [12,14,15].

DPRESS was formulated to address these needs. It is based on two simple assumptions: that the uncertainty in prediction for new objects (e.g., molecular structures) is likely to be dominated by the error in prediction for objects near them in the descriptor space; and that this influence is, to a first approximation, inversely related to the distance between them. The "true" dependence may well be more complex in some cases, but the size of the training set needed to characterize that dependence will almost always be impractically large. In any event, such dependence is likely to reduce to a linear relationship over the relatively short ranges of QSAR extrapolations that have any chance of being relevant.

The fact that the predictive uncertainties derived from DPRESS analysis are sometimes more conservative than necessary for large errors is of some concern, though that is certainly preferable to the alternative of their being overly optimistic; further work in this area is a matter on ongoing investigation. Nonetheless, the method is intrinsically less constraining than the classical quadratic relationship based on distance from the mean (Eq. 4). Given how much predictive error varies across the model space (e.g., Fig. 4), any approach based on the overall *s*_PRED _seems bound to be overly optimistic regarding the reliability of predicted potencies for some compounds.

The underlying QSARs examined here – CoMFA and HQSAR – both rely on (nominally [46]) linear PLS, but there is no intrinsic reason that the method cannot be more broadly applied. The key point is that the error being distributed must be predictive – i.e., it needs to reflect predictions made for objects not included in the training set. LOO cross-validation yields the most information for any given dataset, but a leave-some-out approach should be a viable alternative. The predictive errors obtained from the validation sets often used in ANN analysis could be used as well, since there is no intrinsic reason that a linear model for local error distribution should be incompatible with a QSAR that is non-linear on a global scale.

The usual reasons for preferring LSO over LOO cross-validation are unlikely to be relevant to DPRESS calculations, however. LOO can indeed be distorted when the training set is biased due to redundancy, but DPRESS based on LOO turns out to be conservative in such a situation (see above). The reduction in *s*_CV _that occurs when the sampling density in one particular area of descriptor space is high is reflected in a reduction in the error that each *individual *prediction contributes to the PRESS. But  is not a root mean square, so the effect on its value is offset by the fact that biased sampling necessarily: increases the total number of errors; decreases their spread (*d*_00_); increases the distance between the training set and most new observations; or effects some combination thereof.

Diverse training sets representative of the full structural space produce more reliable local uncertainty estimates than do biased training sets, indicating that taking care to avoid undue sampling bias (redundancy) in the training set is worth the effort. Even the biased training set *R*, however, did better than setting the uncertainty of prediction for a new object equal to the observed error for the closest object in the training set (Fig. 5 and 7). Moreover, the errors falling outside the range expected based for the calculated  for *R *were false positives, the least serious type of error to make when trying to predict toxicity.

There are two fundamental differences between the estimate of predictive uncertainty derived from classical theory (Eq. 4) and the DPRESS model represented by Eqs. 7–9. The first difference is that Eq. 4 is a sum of squares, whereas Eq. 7 is a sum of linear terms. Using a sum of squares formulation was considered for DPRESS, but was found to consistently overestimate the uncertainty of prediction (details not shown). The second difference is that Mahalanobis distances *d *measured in the model space are used in the classical model, whereas Euclidean distances measured in the descriptor space are used in DPRESS. The less parametric approach is followed for DPRESS because the variation in one or more variables in a particular training set may not be large enough to reveal the influence that variable might exert if examined across a greater range. The small coefficient assigned to such a variable in that event means that substantial deviations in its value will have a negligible effect on distances in the model space. Sticking with distances in the "raw" descriptor space rather than using the descriptor weights from **b **to calculate a Mahalanobis distance is more conservative – it assumes that variation in things that have yet to be explored are likely to make predictions less reliable.

## Conclusion

Examination of the distribution of predictive errors across the descriptor space makes it clear that errors are consistently larger in some regions than in others – i.e., the predictive error is heteroskedastic (Fig. 4). Given that a major use of QSAR predictions is in chemoinformatic tabulations used by medicinal chemists and other third parties, it would be good practice to routinely attach some estimate of uncertainty to each prediction. Doing so based on some analytical estimator would be preferable, but is impractical in most real-world situations because it requires detailed *a priori *knowledge of the global dependence of error on the descriptors. In the absence of such knowledge, a locally linear estimator of predictive reliability that is embedded in the sample space represents a reasonable alternative. Partition of predictive error sum of squares (DPRESS) provides just such an estimator in a form – that of a standard error – that is widely understood by those likely to use it. The calculations involved are straightforward and the estimator produced is a qualitatively (Fig. 4 and 6) and quantitatively (Fig. 5 and 7) reliable estimate of how much confidence one should place in the associated prediction. Moreover, though the particular applications studied here involved PLS models built using 2D and 3D descriptors, the technique is likely applicable to any regression method that can be reformulated in kernel-based terms [12,47].

It is also important when constructing the model in the first place to examine the distribution of predictive error in the descriptor space. If uncertainty is homoskedastic, a classical or uniform distribution model may provide a somewhat more precise estimate of predictive uncertainty. Should (e)NLM or principal components analysis (PCA) indicate heteroskedacity, however, a DPRESS calculation should be carried out before applying the model – e.g., for prioritizeing compounds for synthesis, acquisition or detailed testing. DPRESS may also serve to highlight regions of structural space from which more data needs to be gathered.

## Experimental

Ordinary multiple linear regression is not suitable when the number of descriptors in a data set exceeds the number of observations. PLS [4] was used instead, with the appropriate number of latent variables (components) to include (i.e., the model complexity) being the number corresponding to the first minimum in the "leave-one-out" cross-validated standard error (*s*_CV_). This measure of internal consistency is obtained by setting aside each of the *n *compounds in the training subset in turn and trying to predict its activity using the other *n *- 1 compounds in the training set. The external error of prediction (*s*_PRED_) was calculated as the root mean square error for the *N *- *n *compounds left out of the model calculation altogether.

### Training set selection

Boosted training sets were obtained by applying OptiSim selection to the full data set. OptiSim selection entails drawing a series of random subsamples of size *k *from the data set of interest. For each subsample in the series, the individual that is most different from those selected from previous subsamples is extracted and added to the selection set *S*. This procedure results in a representative but diverse selection set that samples the full data set space both efficiently and effectively [42]. Here the structural space was defined in terms of the Tanimoto similarity *T*(*a*, *b*) between the corresponding UNITY substructural fingerprints [48]. The individual *a *in the *i*^th ^subsample for which max(*T*(*a*, *b*): *b *∈ *S*) is smallest was added to *S*. Candidates with a Tanimoto similarity greater than 0.8 to any compound already in the selection set were deemed redundant and were excluded from subsamples.

The selection process was repeated five times with *n *= 75 and *k *= 4, using a different random number seed each time. Five inhibitors appeared in every boosted training set, including the thiophene, cyclopentadiene and isoxazole analogs that fall outside the five major classes. A total of 113 inhibitors were not selected for any of the boosted training sets, whereas 191 were selected for at least one of them.

### Molecular fields

CoMFA involves using PLS to identify correlations of biological activity with variations in steric and electrostatic molecular fields, which requires that the molecules under consideration be put into similar conformations and into a common frame of reference as a key part of the process. Here, conformations were set and molecular structures aligned based on the homologous atoms in their central and peripheral rings, as has been described in detail elsewhere [39]. Charges were calculated using the method of Marsili and Gasteiger [49], as extended in SYBYL [50] to take the distribution of *π *electrons into account ("Gasteiger-Hückel charges"). Coulombic and Lennard-Jones interaction energies were calculated on a 2 Å rectilinear grid extending 4 Å beyond the edge of any molecule in the full data set. The probe atom used to calculate the fields was an *sp*^3^-hybridized carbon monocation. Interaction energies were truncated at nominal values above 30 kcal/mol, and electrostatics were ignored within the steric envelope of each inhibitor.

### Molecular holograms

The first step in constructing a molecular hologram is to identify all substructures in a molecule that fall within a specified size range – here, all fragments made up of four to seven atoms, with hydrogens ignored and bond types taken into account. Each fragment is then mapped into a compressed count vector of specified length using a hashing function, so that the elements of that count vector can be used as descriptors in subsequent PLS analyses [36]. The hashing means that different fragments may map to the same position in the final count vector. The fragments overlap, however, so each substructure contributes to many fragment counts. The result is that the noise introduced by "collisions" for a few subfragments constitutes a relatively minor perturbation that is, on average, self-limiting. Overfit PLS models are characteristically unstable to such perturbations, however, so surveying a range of hash lengths and picking one with good but representative statistical properties is a good way to avoid picking a length whose cross-validation statistics are overly optimistic. This is a non-parametric perturbation analysis analogous to looking at the effect of small perturbations in response to assess model stability [28].

### Visualization

2D depictions of the relationship between different compounds were obtained using the embedded non-linear mapping (eNLM) facility [40] in Benchware DataMiner [51]. "Ordinary" NLM can be thought of as placing springs between all pairs of points in the original descriptor space, then compressing the ensemble into two dimensions in such a way that the residual tension in those springs is minimized. Embedded NLM differs in that parts of springs longer than some specified threshold length (horizon) are treated as elastic to extension, i.e., they do not contribute to the overall stress in the system. Here, spring "tensions" were based on the block-wise autoscaled Euclidean distances ("CoMFA Standard scaling" [32]) between the molecular fields or between the molecular holograms of different compounds.

### DPRESS

CoMFA and HQSAR analyses were carried out in SYBYL. The distances *d*_t, i _used to partition the PRESS were taken from the SAMPLS.dist file generated by the SYBYL interface as input to the SAMPLS program [52] and represent inter-observation distances in the descriptor space after autoscaling has been applied. The descriptors used here are already either fully commensurate (HQSAR) or are piecewise commensurate (within steric and electrostatic fields but not between them, for CoMFA), so "CoMFA standard" (block) autoscaling was used [33]. Observed and predicted responses were taken from the SAMPLS.out file generated by the SAMPLS program.

Localized predictivity estimates were calculated by combining scripts written in SYBYL programming language (SPL) with spreadsheet manipulations carried out in Excel. For each compound *t *in the training set, the scaling factor *γ*_t _was calculated based on the observed predictive variance (squared cross-validation error of prediction, *δ*_i _^2^) for every *other *compound in the training set (*i *≠ *t*) weighted inversely by the square of the Euclidean distance between the two (*d*_t, i_) in the descriptor space (Eq. 8). A normalization factor *α*_i _for each compound *i *was calculated as the sum of squared distances to all other compounds in the training set (Eq. 9). A limiting proximity term of 1/*n *was included to ensure reasonable behavior for closely-spaced compounds where *d*_t, i _approaches 0; this works well when the descriptors have been autoscaled in some way before use.

The individual scaling factors *γ*_t _obtained from the *n *LOO cross-validation errors for the training set were used to calculate an estimate  for the predictive uncertainty associated with each new structure *u *based on the observed cross-validation error of the training set compound (t*) lying closest to it in the descriptor space, its distance from t*, and the scaling factor *γ*_t* _derived from the model cross-validation analyses (Eqs. 7–9).

## Abbreviations

ANN: artificial neural network; BLUE: best linear unbiased estimator; CoMFA: comparative molecular field analysis; CV: cross-validation; *d*: distance; *δ*: predictive error for a compound outside the training set; *e*: residual error for a compound in the training set; eNLM: embedded non-linear mapping; HQSAR: hologram QSAR; LOO: leave-one-out; PCA: principal components analysis; DPRESS: distributed predictive error sum of squares; PLS: partial least squares with projection to latent structures; PRESS: predictive error sum of squares; QSAR: quantitative structure/activity relationship; *s*: standard error for a sample; SPL: SYBYL programming language.

## Competing interests

The author was formerly an employee of Tripos International, which holds exclusive rights to the CoMFA and HQSAR technologies used here to illustrate the use of DPRESS. Tripos provided the SYBYL program to Biochemical Infometrics but did not provide funding for the work described herein.

## References

[B1] Hansch C Maloney PP Fujita T Muir RM Correlation of biological activity of phenoxyacetic acids with Hammett substituent constants and partition coefficients Nature 1962 194 178 180

[B2] Hansch C Fujita T p-*σ*-*π *Analysis. A method for the correlation of biological activity and chemical structure J Am Chem Soc 1964 86 1616 1626

[B3] Jurs PC Chou JT Yuan M Computer-assisted structure-activity studies of chemical carcinogens. A heterogeneous data set J Med Chem 1979 22 476 483 458798 10.1021/jm00191a004

[B4] Wold S Ruhe A Wold H Dunn WJ III The collinearity problem in linear regression. The partial least squares (PLS) approach to generalized inverses SIAM J Sci Stat Comput 1984 5 735 743

[B5] Baumann K Albert H von Korff M A systematic evaluation of the benefits and hazards of variable selection in latent variable regression. Part I. Search algorithm, theory and simulations J Chemometrics 2002 16 339 350

[B6] Baumann K von Korff M Albert H A systematic evaluation of the benefits and hazards of variable selection in latent variable regression. Part II. Practical applications J Chemometrics 2002 16 351 360

[B7] Schultz T Hewitt M Netzeva T Cronin M Assessing applicability domains of toxicological QSARs: definition, confidence in predicted values, and the role of mechanisms of action QSAR Comb Sci 2007 26 238 254

[B8] Giuliani A Benigni R van de Waterbeemd H, Testa B, Folkers G Modeling without boundary conditions: an issue in QSAR validation Computer-Assisted Lead Finding and Optimization 1997 Weinheim: Wiley-VCH 51 63

[B9] Sheridan RP Feuston BP Maiorov VN Kearsley SK Similarity to molecules in the training set is a good discriminator for prediction accuracy in QSAR J Chem Inf Comput Sci 2004 44 1912 1928 15554660 10.1021/ci049782w

[B10] Guha R Jurs PC Determining the validity of a QSAR model – a classification approach J Chem Inf Model 2005 45 65 73 15667130 10.1021/ci0497511

[B11] He L Jurs PC Assessing the reliability of a QSAR model's predictions J Mol Graph Model 2005 23 503 523 15896992 10.1016/j.jmgm.2005.03.003

[B12] Schroeter TS Schwaighofer A Mika S Ter Lakk A Suelzle D Ganzer U Heinrich N Müller K-R Estimating the domain of applicability for machine learning QSAR models: a study on aqueous solubility of drug discovery molecules J Comput-Aided Mol Des 2007 21 651 664 18060505 10.1007/s10822-007-9160-9

[B13] Benigni R Bossa C Predictivity of QSAR J Chem Inf Model 2008 48 971 980 18426198 10.1021/ci8000088

[B14] Tetko IV Sushko I Pandey AK Zhu H Tropsha A Papa E Öberg T Todeschini R Fourches D Varnek A Critical assessment of QSAR models of environmental toxicity against *Tetrahymena pyriformis*: focusing on applicability domain and overfitting by variable selection J Chem Inf Model 2008 48 1733 1746 18729318 10.1021/ci800151m

[B15] Weaver S Gleeson MP The importance of the domain of applicability in QSAR modeling J Mol Graph Model 2008 26 1315 1326 18328754 10.1016/j.jmgm.2008.01.002

[B16] Johnson DE Wolfgang GI Predicting human safety: screening and computational approaches Drug Discov Today. 2000 5 445 454 11018595 10.1016/s1359-6446(00)01559-2

[B17] Bassan A Worth AP The integrated use of models for the properties and effects of chemicals by means of a structured workflow QSAR Comb Sci 2008 27 6 20

[B18] Walker JD Carlsen L Jaworska J Improving opportunities for regulatory acceptance of QSARs: the importance of model domain, uncertainty, validity and predictability Quant Struct-Act Rel 2003 22 6 20

[B19] Snedecor GW Cochran WG Statistical Methods 1989 8 Iowa State Press, Ames, IA

[B20] Kleinknecht RE Error estimation in PLS latent variable structure J Chemometrics 1996 10 687 695

[B21] Denham MC Prediction intervals in partial least squares J Chemometrics 1997 11 39 52

[B22] Faber K Kowalski BR Propagation of measurement errors for the validation of predictions obtained by principal component regression and partial least squares J Chemometrics 1997 11 181 238

[B23] Morsing T Ekman C Comments on construction of confidence intervals in connection with partial least squares J Chemometrics 1998 12 295 299

[B24] Wold S Validation of QSARs Quant Struct-Act Rel 1991 10 191 193

[B25] Clark RD Sprous DG Leonard JM Höltje H-D, Sippl W Validating models based on large data sets Rational Approaches to Drug Design 2001 Barcelona: Prous Science 475 485

[B26] Golbraikh A Tropsha A Beware of q2! J Mol Graph Model 2002 20 269 276 11858635 10.1016/s1093-3263(01)00123-1

[B27] Golbraikh A Tropsha A Predictive QSAR modeling based on diversity sampling of experimental datasets for the training and test set selection J Comput-Aided Mol Des 2002 16 357 269 12489684 10.1023/a:1020869118689

[B28] Clark RD Fox PC Statistical variation in progressive scrambling J Comput Aided Mol Des. 2004 18 7-9 563 576 15729855 10.1007/s10822-004-4077-z

[B29] Hawkins DM Basak SC Mills D Assessing model fit by cross-validation J Chem Inf Comput Sci 2003 43 579 586 12653524 10.1021/ci025626i

[B30] Sutherland JJ O'Brien LA Weaver DF A Comparison of methods for modeling quantitative structure-activity relationships J Med Chem 2004 47 3777 3787 15481990 10.1021/jm0497141

[B31] Bush BL Nachbar RB Jr Sample-distance partial least squares: PLS optimized for many variables, with application to CoMFA J Comput-Aided Mol Des 1993 7 587 619 8294948 10.1007/BF00124364

[B32] Cramer RD III Patterson DE Bunce JD Comparative molecular field analysis (CoMFA). 1. Effect of shape on binding of steroids to carrier proteins J Am Chem Soc 1988 110 5959 5967 22148765 10.1021/ja00226a005

[B33] Cramer RD III DePriest SA Patterson DE Hecht P Kubinyi H The developing practice of comparative molecular field analysis 3D QSAR in Drug Design: Theory, Methods and Applications 1993 Leiden: ESCOM 443 485

[B34] Kroemer RT Hecht P Guessregen S Liedl KR Kubinyi H, Folkers G, Martin YC Improving the predictive quality of models 3D QSAR in Drug Design 1998 3 Dordrecht: Kluewer/ESCOM 41 56

[B35] Heritage TW Lowis DR Parrill AL, Reddy MR Molecular Hologram QSAR Rational Drug Design: Novel Methodology and Practical Applications, ACS Symposium Series 719 1999 Washington DC: American Chemical Society 212 225

[B36] Tong W Lowis DR Perkins R Chen Y Welsh WJ Goddette DW Heritage TW Sheehan DM Evaluation of quantitative structure-activity relationship methods for large-scale prediction of chemicals binding to the estrogen receptor J Chem Inf Comput Sci 1998 38 669 677 9722424 10.1021/ci980008g

[B37] Seel M Turner DB Willett P Effect of parameter variations on the effectiveness of HQSAR analyses Quant Struc-Act Rel 1999 18 245 252

[B38] Chavatte P Yous S Marot C Baurin N Lesieur D Three-dimensional quantitative structure-activity relationships of cyclooxygenase-2 (COX-2) inhibitors: a comparative molecular field analysis J Med Chem 2001 44 3223 3230 11563921 10.1021/jm0101343

[B39] Clark RD Boosted leave-many-out cross-validation: the effect of training set and test set diversity on PLS statistics J Comput-Aided Mol Des 2003 17 265 275 13677492 10.1023/a:1025366721142

[B40] Clark RD Patterson DE Soltanshahi F Blake JF Matthew JB Visualizing substructural fingerprints J Mol Graph Model 2000 18 404 411 11143558 10.1016/s1093-3263(00)00065-6

[B41] Agrafiotis DK Stochastic Proximity Embedding J Comput Chem 2003 24 1215 1221 12820129 10.1002/jcc.10234

[B42] Clark RD OptiSim: an extended dissimilarity selection method for finding diverse representative subsets J Chem Inf Comput Sci 1997 37 1181 1188

[B43] Clark RD Langton WJ Balancing representativeness against diversity using optimizable *K*-dissimilarity and hierarchical clustering J Chem Inf Comput Sci 1998 38 1079 1086

[B44] Clark RD Getting past diversity in assessing virtual library designs J Brazil Chem Soc 2002 13 788 794

[B45] Clark RD Shepphird JK Holliday J The effect of structural redundancy in validation sets on virtual screening performance J Chemometrics

[B46] Kim KK Kubinyi H Nonlinear dependence in comparative molecular field analysis 3D QSAR in Drug Design Theory, Methods and Applications 1993 Leiden: ESCOM 71 82 10.1007/BF00141576 8473919

[B47] Embrechts MJ Szymanski B Sternickel K Ovaska SJ Introduction to scientific data mining: Direct kernel methods and applications Computationally Intelligent Hybrid Systems: The Fusion of Soft Computing and Hard Computing 2005 New York: Wiley 317 362

[B48] Haranczyk M Holliday J Comparison of similarity coefficients for clustering and compound selection J Chem Inf Model 2008 48 498 509 18293953 10.1021/ci700413a

[B49] Gasteiger J Marsili M Iterative partial equalization of orbital electronegativity – a rapid access to atomic charges Tetrahedron 1980 36 3219 3228

[B50] SYBYL, v 8.0 2008 Tripos International: St. Louis, MO

[B51] Benchware DataMiner, v. 1.6 2007 Tripos International: St. Louis, MO

[B52] Bush B SAMPLS: SAMple-driven Partial Least Squares 1993 Merck & Co., Inc.: Rahway, NJ

